# Embedding Scientific Communication and Digital Capabilities in the Undergraduate Biomedical Science Curriculum

**DOI:** 10.3389/bjbs.2023.11284

**Published:** 2023-04-19

**Authors:** Beverley C. Millar, Andrei Tarasov, Nigel Ternan, John E. Moore, Colette Murphy

**Affiliations:** ^1^ School of Biomedical Sciences, Ulster University, Coleraine, United Kingdom; ^2^ Northern Ireland Public Health Laboratory, Belfast City Hospital, Belfast, United Kingdom; ^3^ Centre for Higher Education Research and Practice, Ulster University, Belfast, United Kingdom

**Keywords:** biomedical science, lay summary, scientific communication, visual abstract, digital capabilities, curriculum, reflection

## Abstract

**Introduction:** Scientific communication, particularly the dissemination of research findings to both the scientific community and the general public, are skills required of graduates embarking on post-graduate studies and employment within the biomedical sciences sector. The aims of this action research project were to i) co-design an online scientific communication and digital capabilities resource, constructively aligned to the learning objectives of a final year undergraduate investigative research project; ii) ensure resource flexibility for future adaptation by others iii) embed authentic scientific communication learning assessments, namely, the preparation of a lay summary and visual abstract and iv) promote students’ awareness of developed digital capabilities and transferable skills through written reflection.

**Materials and Methods:** Student engagement, self-efficacy, experiences and performance and staff perceptions (*n* = 15) were evaluated by a mixed methods approach. Qualitative data was gathered from focus sessions, free text responses within questionnaires and content analysis of students’ written reflections (*n* = 104). Quantitative data from 5-point Likert responses within student questionnaires (*n* = 31) and analysis of student scientific and lay writing (*n* = 146) using the readability parameters Flesch-Kincaid Grade Level and Flesch Reading Ease were analysed using non-parametric statistical methods.

**Results:** A learning resource was co-designed with students, staff, local, national and international contributors and valued by both students and staff, enabling students to prepare scientific communication outputs of a professional standard by application of digital, analytical and scientific communication skills. Students prepared lay summaries which were statistically (*p* < 0.0001) more readable than their paired scientific abstracts. Significant correlations between easier readability of lay summaries and awarded marks for the written elements of the module were noted. Students reported their digital and communication capabilities increased significantly (*p* < 0.0001) throughout, from limited to good/excellent and reflected on the numerous transferable skills developed during preparation of assessments, with 75% reflecting on their digital capabilities.

**Discussion:** Undergraduate students developed, appreciated and used varied scientific communication and digital skills to articulate research findings. The embedding of such activities throughout all levels of higher education will enable students to develop their digital and scientific skills and reflect on the development of such transferable skills for application in their future careers.

## Introduction

Pivotal to the effective and inclusive delivery of healthcare, is the ability to draw upon scientific evidence to inform healthcare practice as well as communicate with varied stakeholders and patients. Effective communication is a skill which all employers seek irrespective of the type of employment or the role an employee fulfils within the employer’s institution. Within the healthcare sector effective communication is an essential competency which the regulatory body, Health and Care Professions Council (HCPC), insists that healthcare professionals, including biomedical scientists, must meet in order to fulfil their Standards of Proficiency, which have been modified and are effective from 1 September 2023 (1). These modified standards reinforce the importance that healthcare professionals possess essential verbal and non-verbal skills to engage and effectively communicate with multi-disciplinary members of the professional healthcare team as well as patients. Furthermore, these modified standards of proficiency now place greater emphasis on registrants using information, communication and digital technologies to communicate effectively (1). Such skills will aid to ensure that factors such as age, capacity, learning ability and physical ability are considered to ensure inclusiveness in order that service users and their carers can make informed decisions on the current information or evidence available (1).

Lay communication is important for several reasons, namely, in relation to public awareness and engagement, trust in scientific research, influencing public behaviours and opinions, improving scientific and health literacy, recruitment in clinical trials, as well as political and funding support (2, 3). Additionally, Editors of peer-reviewed journals have focused on innovative modalities to disseminate research digitally and the visual abstract provides one such approach to convey research findings visually and succinctly (4). Digital literacy is of fundamental importance in enabling the successful development of scientific communication competencies, both in relation to digital technical skills, as well as encouraging a positive approach to utilising these skills within a varied employment sector (5, 6).

Central to biomedical science undergraduate research programmes are final year research/investigative projects which promote the development of practical research skills including technical, experimental design, data acquisition, analytical and problem-solving skills. The professional body, the Institute of Biomedical Science (IBMS) which accredits these undergraduate degree programmes (7) and the recent Quality Assurance Agency (QAA) for Higher Education Benchmark statement for Biomedical Sciences (8) have set the requirement that the development of key transferable skills should be encouraged including, competency in a range of appropriate communication platforms, both digital and physical, for the effective dissemination of information to scientific and lay audiences. The QAA Benchmark also states that authentic assessment in Biomedical Science degree programmes should include various types of communication, e.g., graphical, posters, video, website and written formats targeting a varied audience (8). It is therefore important that students comprehend the various implications of research findings and be taught why and how the significance of these findings are disseminated, to both the scientific community and the general public who have varied levels of understanding (9).

An engaging innovative curriculum designed as per the Integrated Curriculum Design Framework (10) and underpinned by pedagogical methods would ensure a focus on the development of such scientific communication skills and associated digital capabilities thereby enhancing the student experience. An online resource developed with students and staff in collaboration and partnership with other related professional communities would support students prepare learning/assessment activities enabling the development of higher order critical thinking skills and communication competencies required by employers, including those within the healthcare and scientific sectors.

The aims of this action research project (ARP) were to i) co-design an online scientific communication and digital capabilities resource, constructively aligned to the learning objectives of a final year undergraduate investigative research project; ii) ensure resource flexibility for future adaptation by others iii) embed authentic scientific communication learning assessments, the preparation of a lay summary and visual abstract and iv) promote students’ awareness of developed capabilities and transferable skills through written reflection.

The objectives of this ARP were to i) measure the extent to which students utilised the co-produced online resource, ii) measure the effectiveness of a standardised approach to assessing the students’ professionalism and skills development *via* two novel learning activities, namely the preparation of a lay summary and visual abstract, iii) evaluate student perceptions of how their confidence, competence and capabilities were developed through the scientific communication and digital skills learning activities and iv) evaluate the potential of the shared resource to be modified and used in other levels of teaching and assessment.

## Materials and Methods

### Participants

All undergraduate students (*n* = 148) enrolled the final year undergraduate research project 60 credit point module during the academic year 2020–21, within the School of Biomedical Sciences at Ulster University were invited to participate in this study. Students who completed the module (*n* = 146) in the normal timeframe were enrolled in different Honours degree programmes, namely Biomedical Science 3y programme (*n* = 79), Biomedical Science Diploma in Professional Practice (DPP) (Pathology) (*n* = 20), BMS Diploma in Professional Practice (*n* = 23) and Biology (*n* = 24). All Biomedical Science courses were accredited by the IBMS. All supervisory academic staff (*n* = 38) associated with the assessment of submitted investigative dissertations were invited to complete a survey as detailed below.

### Evaluation Methodology

A mixed methods approach was used to evaluate this ARP. Qualitative data gathered through reflective feedback, focus groups and free text responses within student and academic staff questionnaire responses were used to evaluate the intervention in terms of’ perceptions and experiences. Quantitative data and statistical evaluation allowed further refining and evaluation of the outcomes of this project from data gathered through questionnaires and analysis of lay-writing outputs (see below) (11).

### Student Focus Groups

An e-mail was sent to all students enrolled in investigative project module from the School office calling for expressions of interest, to contribute to an online focus group to co-design resources to support students prepare novel scientific communication assessments and highlight the importance of transferable employability skills. A virtual meeting was held using the web-based virtual learning environment and learning management system, Blackboard Learn, by means of the online meeting tool, BBL Collaborate Ultra. Five self-nominated students, a Visiting Professor from the healthcare sector, with supervisory responsibilities within the Final Year Investigative Project module and lead study author were in attendance. Students outlined the key materials they felt would be required to successfully complete the novel learning activities.

Following completion of all module assessments for the academic year 2020–21, the School e-mailed invitations to all students enrolled in the investigative project module, seeking expressions of interest to participate in an online reflective focus group. Due to availability, two such sessions were held with three students and two staff members in attendance in each session. One session was attended by three students who also attended the initial co-design focus session. All students who expressed an interest in being involved in any of the focus sessions participated in their requested focus group.

### Student Engagement With Online Resource

The online scientific communication and digital capabilities “toolkit” resource was housed on the Blackboard Learn module site. Blackboard Learn statistics tracking enabled an analysis of student access including time periods and frequency of consultation of the online resources.

### Surveys

Following completion of all module assessment for the academic year 2020–21, the School e-mailed invitations, containing a link to respective questionnaires managed through Microsoft Forms, to all students enrolled in the final year research program module (Supplementary Table S1), and supervisory staff (Supplementary Table S2). A single reminder was sent to students 4 weeks after the initial email after the final year examination period.

The student questionnaire provided students the opportunity to qualitatively reflect on the suitability of the resources provided and the acquisition of transferable skills. Using a 5-point Likert Scale, students (*n* = 31 respondents) quantitatively evaluated: i) the development of their capabilities in relation to the assessment tasks and digital literacy; ii) the support provided throughout the module; iii) the importance of embedding transferable skills development in undergraduate degree programmes; iv) their confidence in applying such developed skills in future studies and/or career and v) their preferences on how information and guidance should be delivered.

The staff questionnaire provided staff the opportunity to qualitatively reflect on the introduction of these new assessment activities. Using a 5-point Likert Scale, staff (*n* = 15 respondents) quantitatively evaluated: i) the importance embedding transferable skills development in undergraduate degree programmes; ii) the applicability of embedding the preparation of visual abstracts and lay summaries into the module and iii) how the introduction of these tasks helped in their assessment of students.

### Readability Analyses

The readability of students’ scientific abstracts (*n* = 146) and paired lay summaries (*n* = 146) were analysed using the subscription software package, Readable (www.readable.com). The readable package was chosen as it is reliable, easy to use and widely available (12). Two readability measures, the Flesch-Kincaid Grade Level and the Flesch Reading Ease were used to assess whether the students adapted their writing in consideration of a lay audience. These readability measures were chosen as they have been used widely used and accepted by scientific and non-scientific communities alike (13).

### Qualitative Data Content Analysis

Students were requested to reflect on their experiences during the completion of the Final Year Investigative project, with a particular focus on employability as outlined in the toolkit. A content analysis was performed on the reflective writing of each student who gave signed consent (*n* = 104/146; 71.2%) (14). Content analysis is a recognised qualitative approach to analysing data in pedagogical action research studies and guidance on this thematic analysis approach has been provided by Lin Norton (14). In the case of this study, thematic analysis was based on analysis of the students’ reflective text, namely, categories were constructed focusing on key 21st Century skills namely (ii) foundational literacies (ICT literacy, scientific knowledge, scientific communication literacy), competencies (critical thinking/problem solving, creativity, communication, collaboration/teamwork) and character qualities (independence, flexibility, time management, organisation) as categorised by the World Economic Reform (15). The ICT literacies were further categorised in relation to the six digital capabilities as defined by JISC (16). Following dissection of the students’ reflective writing, a percentage referenced to each category was calculated.

### Statistical Analyses

Data gathered from questionnaire responses and readability analyses (mean ± standard error of the mean) were reported. Statistical analyses were performed using non-parametric methods. For all data, a Kolmogorov-Smirnov test for normality was conducted prior to a Wilcoxon signed-rank test for related groups which were not normally distributed. IBM-SPSS Statistics version 26 was used to perform a one-way analysis of variance to examine for significant differences between courses and a Pearson correlation to examine correlations between readability and awarded assessment marks. Statistical significance was set at *p*=<0.05.

## Results

### Development of the Online Toolkit

**FIGURE 1:**
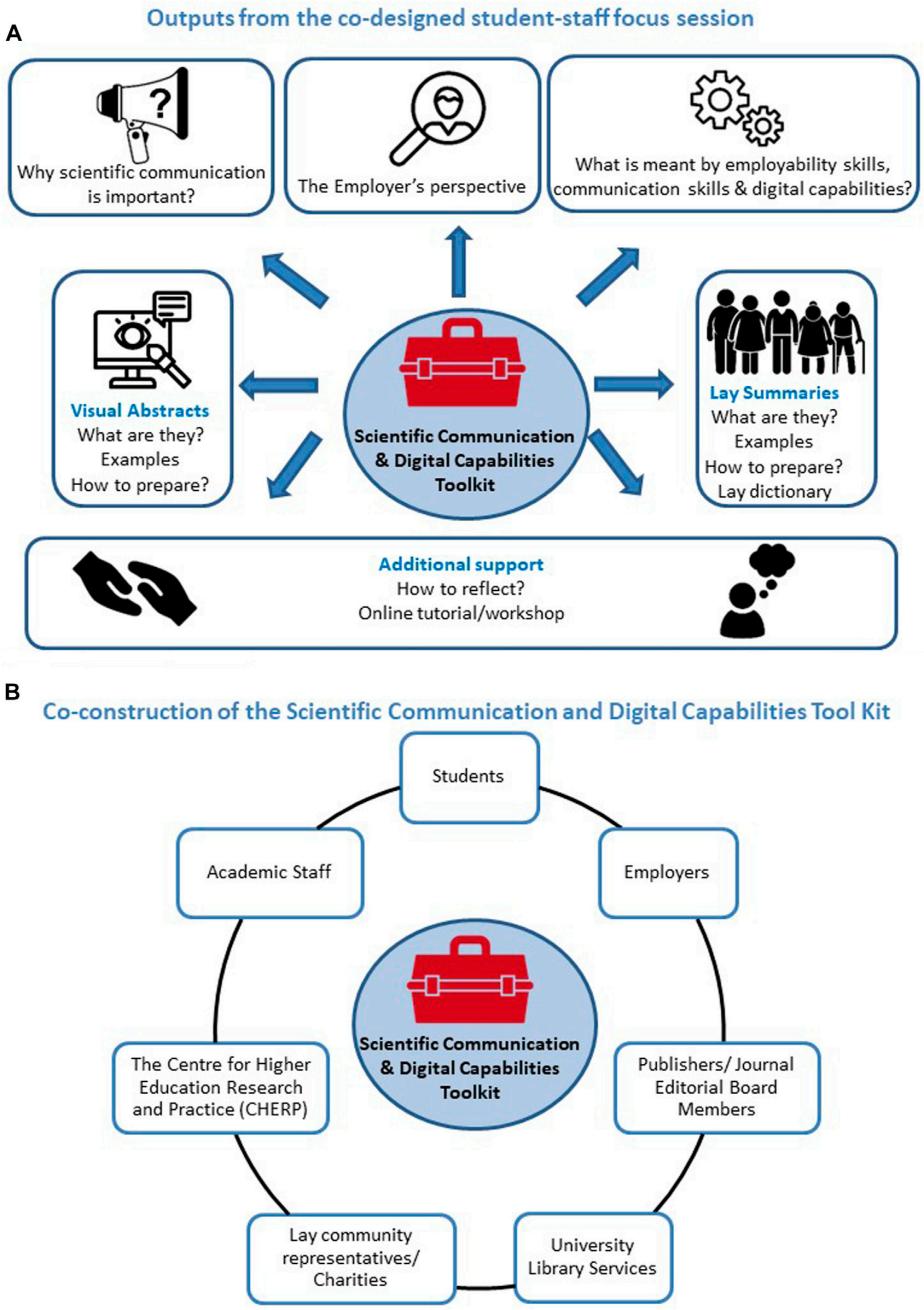
The blueprint of the online Scientific Communication and Digital Capabilities Toolkit prepared following the co-design focus session **(A)** and the various contributors **(B)**.

**FIGURE 2:**
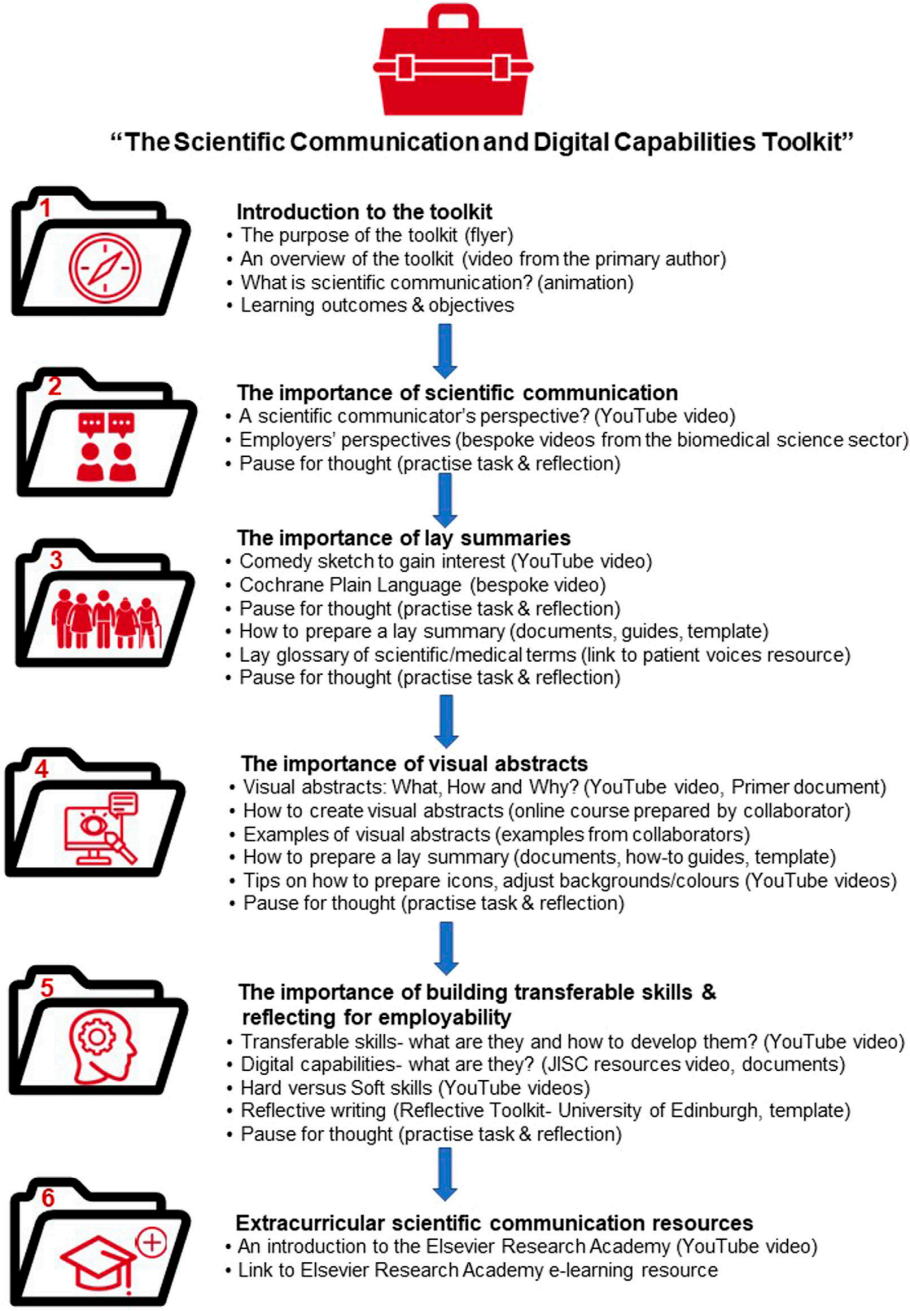
Structure of the scientific communication and digital capabilities tool kit.

During the co-design focus session, students outlined the key materials they felt would be required to successfully complete the learning activities and a blueprint was constructed (Figure 1A). Subsequently, other stakeholders/contributors, internally, locally, nationally and internationally, were contacted and through active participation they contributed to the further design and creation of the educational resource, ensuring that the students’ perspectives were central to this co-produced toolkit (Figure 1B, see Figure 2 for toolkit structure and Supplementary Figure S1 for key public domain resources). Various narrative, interactive, communicative, adaptive and productive media forms, as classified by Laurillard (17) were utilised to provide varied, engaging and informative learning experiences (Table 1).

**TABLE 1:** Examples of technologies used within the online toolkit.

Media form	Technology	Example	Learning experience
Narrative	Text	-Top tips- lay writing	**Apprehending** content shown, told or read
Explain	PowerPoint	-Plain Language (Cochrane presentation)	
Demonstrate	Video	-*How to videos?* remove backgrounds,	
Describe		change icon colour in PowerPoint	
		-*What are videos*?	
		-digital capabilities,	
		-transferable, 21st century & soft skills	
		-Employer’s perspectives	
Interactive	Online tasks	Short courses	**Investigating/exploring** resources, digital tools
Facilitation of reflection, encourage further exploration		-Elsevier Research Academy	
		-Visual Abstracts (Duke University)	
Communicative	Online tutorial	Question and answer session	**Discussing** with student peers and staff
Facilitation of exchanges between students, staff/students			
Adaptive	Feedback	Responding to feedback	**Experimenting**
Facilitation of practise and experimentation		- supervisor/author of toolkit	
		- self-reflection	
Productive Learners demonstrate their understanding	Preparation of assessment communication and reflective outputs	-Tasks associated with each learning resource to encourage active engagement	**Articulating/expressing** what has been learnt
		-Assessed scientific communication outputs	

### Student Engagement With Online Resource

From the statistical report within BBL it was observed that 25% of students (37/148) accessed the toolkit within the first 4.5 h following an announcement of its release, with 35.8% (53/148) having accessed it following 2 weeks of release. Further students accessed the tool kit for the first time in subsequent 3 months (18.2%, 34.5% and 5.4% respectively. Nine students (6.1%) never accessed the toolkit (Supplementary Figure S2). Following an online “drop-in session” on 12 April, which provided students the opportunity to discuss with their peers and tutor the content of the toolkit and any issues which they had in relation to the preparation of the lay summary and visual abstract, there was a renewed interest in the toolkit. Highest activity was noted during the final week prior to submission of assessment materials (Supplementary Figure S3). Various sections of the tool kit were accessed more frequently than others (Table 2), namely those areas which provided specific instructions on how to complete the assessed tasks.

**TABLE 2:** Percentage of students (*n* = 148) who accessed different media formats within the toolkit.

Topic	Format	Details	Access (%)
Introduction	Animated video	*“Scientific communication and digital capabilities toolkit*”-An introduction	88.0
	Videos	The employers’ perspective	15.8
Lay writing	PowerPoint/video	*“An introduction to communicating healthcare research in plain language*”	61.6
	Word document	*“Tips on how to write a lay summary”*	61.6
Visual Abstracts	Short online course	*“How to create effective visual abstracts”*	69.2
	YouTube video	*“How to remove a background from a picture in PowerPoint”*	71.2
Transferable Skills	Web resource	*“What is digital capability?”* (JISC document)	17.1
	YouTube video	“*Transferable Skills –What are they and how can you develop them?”*	17.8
	YouTube video	*“What are digital capabilities?”*	17.8
	Online toolkit	*“Reflective Toolkit”*	93.2

### Surveys

The uptake rate of the student survey was 20.9% (31/148 students), with students enrolled in course programmes as detailed in Supplementary Table S3. The percentage of students who ranked the level of support provided in relation the preparation of the new assessment tasks as good/excellent, in the case of the visual abstract (84%) and lay summary (83%), was higher than other historically embedded resources relating to searching (42%) and reading/analysis (50%) of scientific literature, preparation of a scientific abstract (70%), written paper (70%) and poster (60%), (Supplementary Figure S4). This highlights the importance of student co-development of educational resources to ensure that educational learning resources are optimal to enhance student engagement and facilitate learning.

Students had highest preference for PowerPoint/with voice over for the delivery of educational materials, with least preference attributed to discussion forums (Supplementary Figure S5). The findings shown in Supplementary Figure S5 are important to consider when developing further educational resources or adapting the toolkit for other student cohorts, particularly when such resources are provided online or *via* a blended learning approach (18).

**FIGURE 3:**
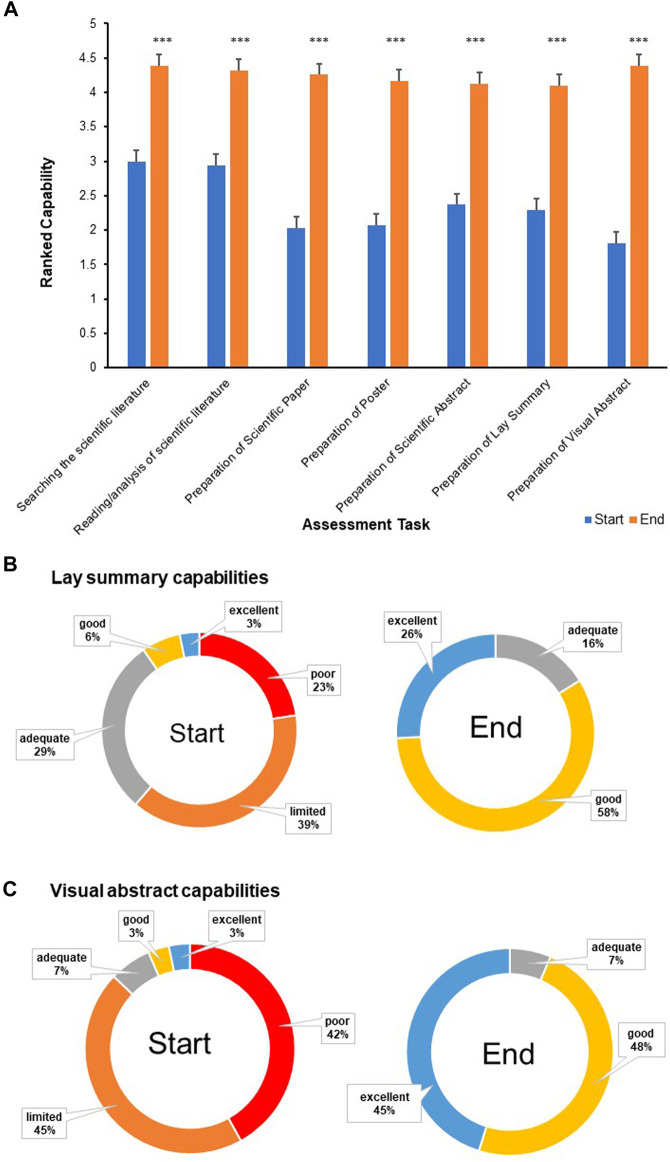
**(A)** The development of students’ capabilities (*n* = 31) (as ranked using the Likert scale 1 = poor 2 = limited 3 = adequate 4 = good 5 = excellent) mean values; ***p < 0.0001. A comparison of the development of students (*n* = 31) capabilities in relation to the preparation of **(B)** Lay Summaries and **(C)** Visual Abstracts.

**FIGURE 4:**
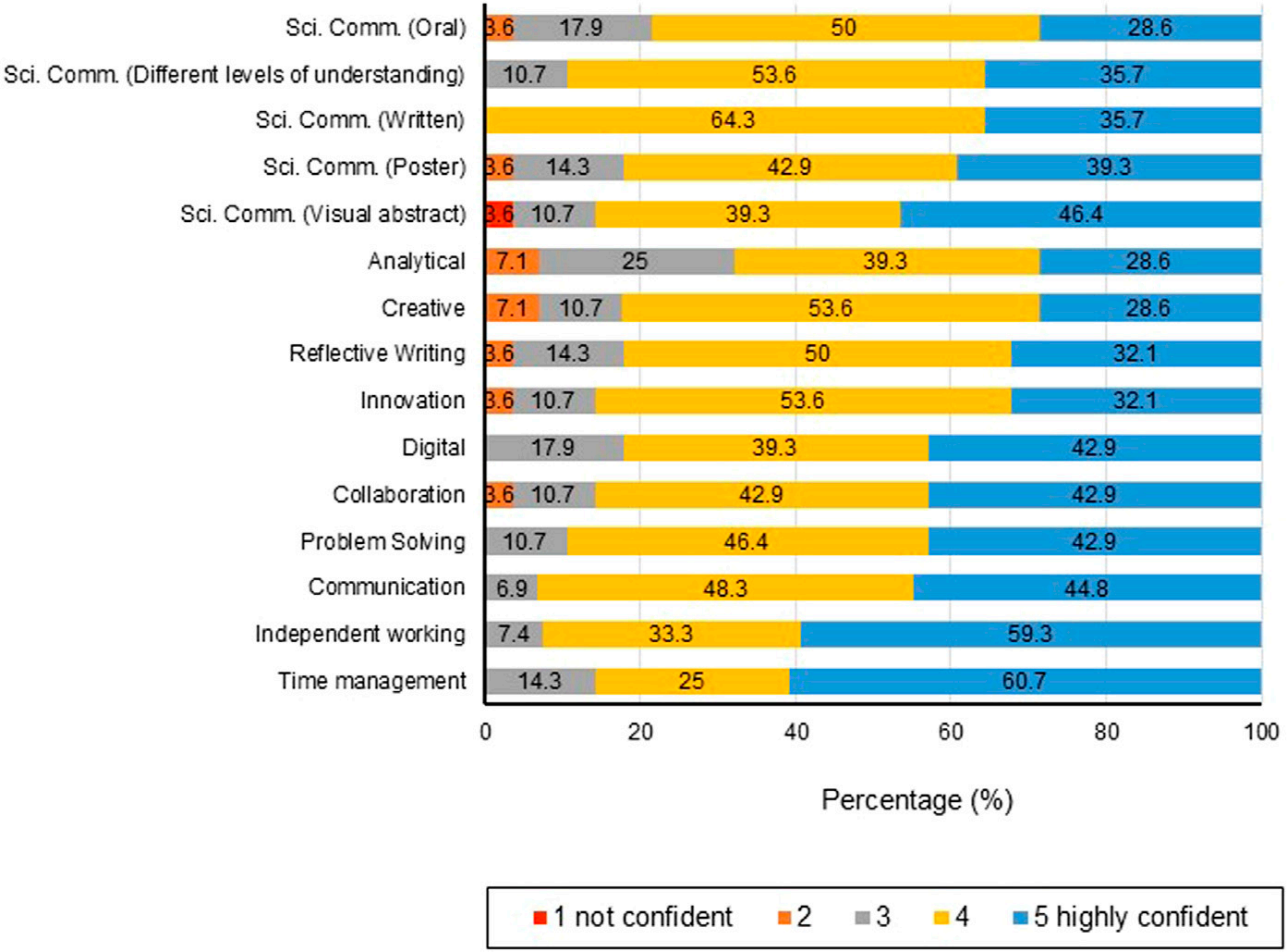
Students’ (*n* = 31) confidence in relation to skills acquired and developed during the investigative research project module.

In terms of self-efficacy, students reported a significant increase in their capabilities in all research focused elements of the investigative project (Figure 3A) In relation to preparing a lay summary, 62% of students stated a low rating (poor/limited) on commencement with a statistically improved higher rating (good/excellent) in 84% of students on completion (Figure 3B). Similarly for the preparation of visual abstracts (87%, poor/limited at commencement and 93%, good/excellent on completion (Figure 3C). Students ranked their confidence in applying developed skills in the future, highly (4–5) in the case of lay scientific communication (89.3%), visual abstract preparation (85.7%) and reflective writing (82.1%) (Figure 4).

Fifteen members of academic staff who supervised and assessed students enrolled in the Investigative Final Year Project module completed the staff survey (uptake rate of 39.5% (15/38)). On analysis of free text provided in submitted questionnaire responses, in relation to the novel scientific communication assessments, overall, staff felt that for undergraduate students and further application in graduate careers, the creation of a lay summary was more applicable than the creation of a visual abstract. In the case of the visual abstract, these included analysis, synthesis and summarising of complex scientific approaches and research findings to formulate key take home messages in a simplified, creative and impactful visual presentation by employing a variety of digital skills. In the case of the lay summary, skills included critical thinking regarding real-world application of their research and awareness of how to express and communicate science using simple language to different stakeholder audiences. Staff reported the visual abstract (46%) and lay summary (53%) were of value when assessing the students they supervised and they helped in the understanding of projects which they marked but did not supervise, visual abstract (57%) and lay summary (61.5%).

**FIGURE 5:**
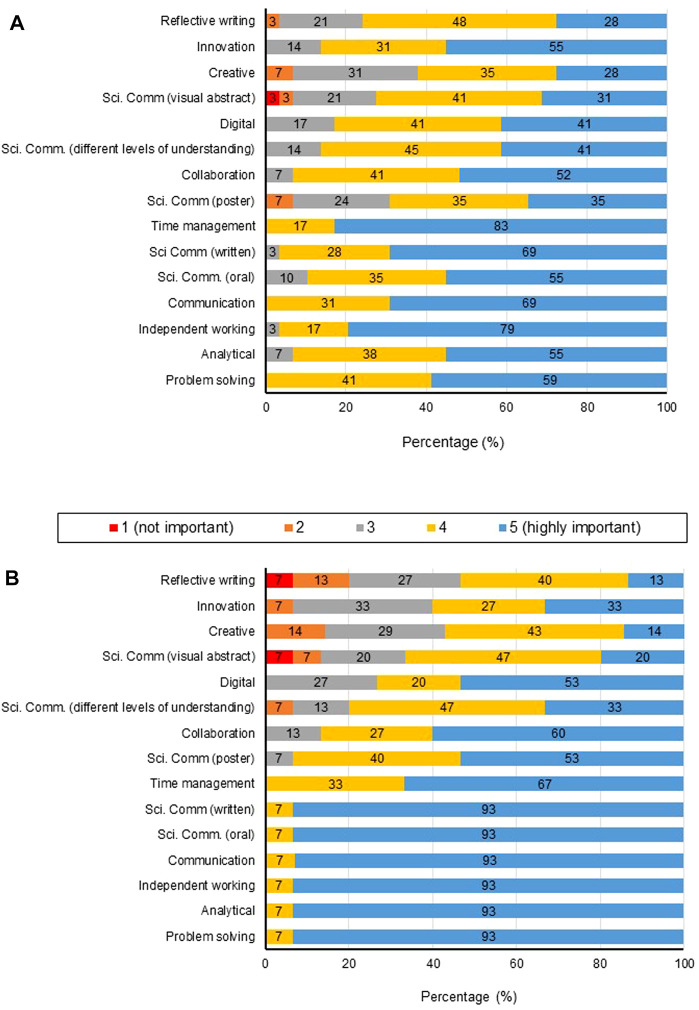
Student (*n* = 31) **(A)** and staff (*n* = 16) **(B)** perspectives on the importance of undergraduate students studying Biomedical Sciences/Biology to have opportunities to develop skills.

In responses to questionnaires, students and staff ranked the importance of having opportunities to develop skills within the undergraduate Biomedical Science courses (Figures 5A, B, respectively).

### Qualitative Data Content Analysis

**FIGURE 6:**
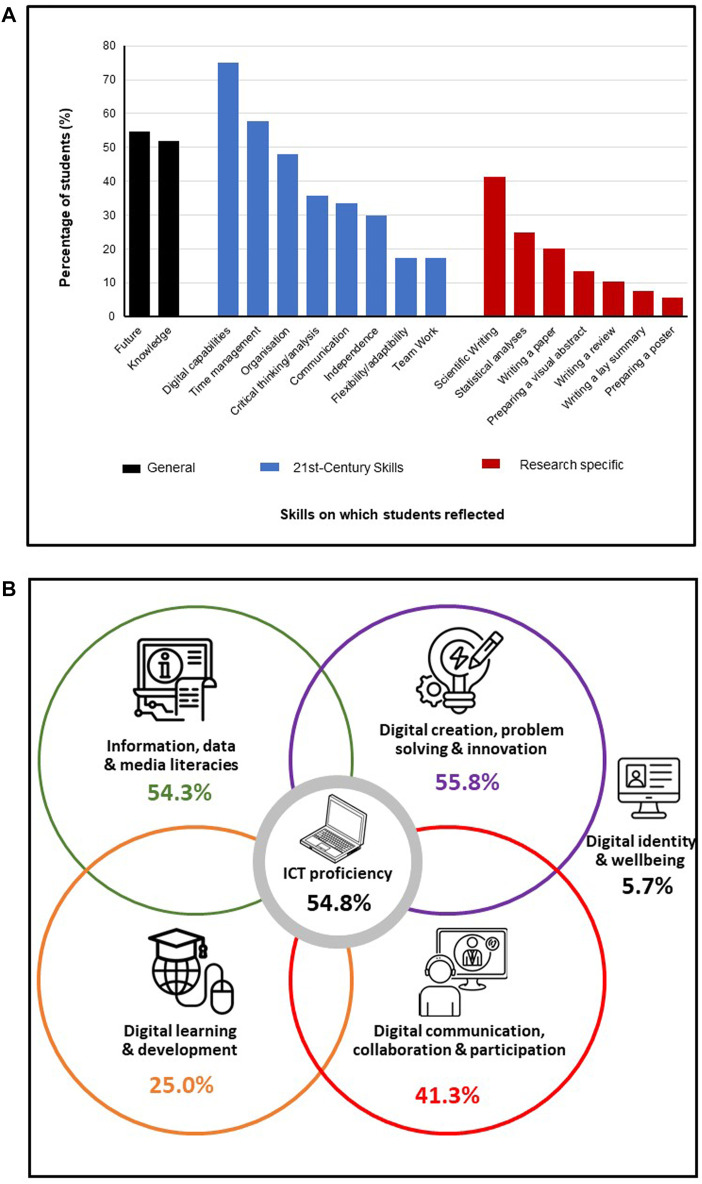
Analysis of student reflections (*n* = 104) on **(A)** 21st Century skills developed as defined by the World Economic Forum, 2015 (15) and **(B)** digital capabilities as classified by JISC (16).

Written reflections (104/146 students; 71.2%) analysed by means of content analysis revealed that students specifically commented on the knowledge acquired throughout the module (51.9%) and the fact that skills acquired will be used in their future career (54.8%). A large proportion of students reflected on a wide variety of subject specific and 21st Century skills which they had developed as shown in Figure 6A.

Further analysis of the digital capability skills acquired indicated that students had an awareness of all of the six digital capabilities to varying degrees, with a primary focus on information, data and media literacies, ICT proficiency, digital creation and digital communication (Figure 6B. One quarter of students used digital formats in relation to self-directed learning, particularly in relation to statistical analyses and bioinformatics. Only 5.7% of students acknowledged the importance of digital wellbeing (Figure 6B), highlighting the importance to embed such awareness within the curriculum, particularly with increasing teaching and assessment delivered either fully online or by a blended learning approach.

### Readability Analyses

Readability metrics of the lay and paired scientific abstracts prepared by the students (*n* = 146) is shown in Table 3 which is compared with the readability of scientific abstracts and paired lay summaries prepared by scientists published in the *Journal of Cystic Fibrosis* and its sister lay journal CF Research News (19). The Flesch Reading Ease (FRE) and Flesch-Kincade Grade Level (FKGL) relating to the student lay summaries were statistically (*p* < 0.0001) higher and lower respectively than their paired scientific abstract indicating improved readability characteristics. There were no significant differences between the students enrolled in the different courses in relation to the readability of either the lay summary or scientific abstract (Supplementary Figure S6). There was a small but significant negative Pearson correlation between the lay FKGL and the marks awarded for the project components (review, dissertation, supervisor’s mark and poster), which indicates there was a correlation between higher marks being awarded to students who demonstrated the skills to successfully moderate their writing for the lay audience. In contrast a statistical negative correlation was only observed in relation to the FRE and supervisor’s mark, when considering the ability to write a scientific abstract for a specialist audience (Supplementary Figure S7).

**TABLE 3:** Readability analysis of student scientific abstracts (*n* = 146) and lay summaries (*n* = 146) compared to those published in a scientific journal and lay sister journal (19).

Target		Flesch Reading Ease (FRE) (mean ± SEM)	Median	Range
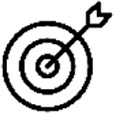	Scientific abstract range 15-18			
	Lay summary 60			
Scientific abstract (Student)		20.3 ± 1.1	20.7	−57.9–45.8
Lay summary (Student)		47.1 ± 1.0***	47.0	−4.1–74.1
Scientific abstract (Journal)		25.2 ± 1.1	25.9	−5.0–56.2
Lay summary (Journal)		43.3 ± 1.0	43.9	11.4–43.9

Target		Flesch-Kincaid Grade Level	Median	Range
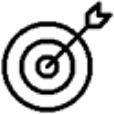	Scientific abstract^a^: range 12.6-25.6			
	Lay summary: 8			
Scientific abstract (Student)		16.4 ± 0.3	16.1	10.9–50.1
Lay summary (Student)		10.7 ± 0.2***	10.8	6.2–20.6
Scientific abstract (Journal)		14.1 ± 0.2	14.0	9.2–8.4
Lay summary (Journal)		11.7 ± 0.1	11.6	8.1–16.3

^a^ (20).

***p < 0.0001; Wilcoxon-signed rank test of student lay summary versus student scientific abstract.

## Discussion

### The Development of Online Resources

As student and staff participants were central to this action research project, a staff-student collaboration approach was used to enable students to be “*proactive, enquiring and productive participants in the learning process*” by co-designing a knowledge-based resource (21). Such engagement builds students’ trust, respect and confidence whilst enabling staff to critically evaluate the feasibility of maintaining the implementation of novel teaching methods within the current module, as well as assessing the validity of embedding such evidence-based pedagogic practice throughout various levels of undergraduate and post graduate curricula in a spiral learning approach (20). The co-design of this toolkit fostered a positive and valued relationship between staff and student, as highlighted in the reflective focus groups. Such a co-design approach should be considered by all staff when developing such teaching and assessment activities, as student outcomes, namely partnership, increased engagement, motivation, ownership, meta-cognitive learning and awareness of the need for the development of transferable skills for employability, as has also been noted by Mercer-Mapstone et al. (22).

An online scientific communication and digital capabilities toolkit was prepared to support students prepare three outputs, namely a visual abstract, lay summary and written reflection focusing on educational experiences within the module and employability. The structure and content of the toolkit, although initially prepared for final year undergraduate students, was not too prescriptive to ensure future adaptability, flexibility and scalability, thereby enabling other teaching staff and users to customise and repurpose for their individual teaching needs. Subsequently, the toolkit was successfully embedded in both MSc and PhD programmes as well as aspects of the toolkit embedded throughout all levels of undergraduate degree programmes.

Central to the design of the toolkit was consideration of all three curricular domains; i) the knowledge of the importance of scientific communication and approaches used; ii) the skills which are required to prepare communication outputs for varied audiences and iii) attributes required to communicate effectively, all of which follow the basis of the “Know/Be/Do framework” (23–25). A significant emphasis was placed on the skills domain, with an overlap between all three domains. How to reflect for employability having completed assessment tasks associated with the module, was embedded to ensure that students had an appreciation of their personal development in relation to attributes and skills required for their future career and employment (10).

The construction of the online resource was underpinned with pedagogical approaches which closely aligned with Gagne’s neo-behaviourist’s theory of hierarchical learning (26). Animation, a comedy sketch and videos from employers’ perspectives initially facilitated, encouraged and motivated students to engage with the resource prior to learning. The resource could be viewed in its entirety and was constructively aligned with the module learning outcomes and learning objectives (27) and was presented with a logical flow in relation to structure and content (Figure 2, Supplementary Figure S1) to ensure ease of navigation through the various detailed layers. Content was provided by authoritative contributors with professional working examples, key tips, “how to” videos and templates. Students were challenged to demonstrate their understanding of the content as they progressed through the learning resource by short “*pause for thought*” self-evaluation and reflective activities. Such self-directed learning coupled with an online tutorial session allowed students to discuss and receive feedback in relation to their approaches to the assessment tasks and subsequently apply what they had learnt to experience creative discovery when completing the final assessment tasks. Various methods of flipped (28) and active learning (29) were facilitated by the resource, namely learning by i) acquisition of content *via* videos, documents and images; ii) enquiry *via* online courses/activities and iii) ultimately production a final creative output (30, 31), thereby applying of all levels of Anderson and Krathwohl’s revised Bloom’s cognitive taxonomy (32).

It must also be considered that the learning styles and approaches used by students of different generations are constantly evolving. Current focus is on the new generation of *Millennials*, the *Centennials,* who are believed to embrace direct involvement in learning through a multimodal approach. Their participation in learning is by *doing* rather than solely *receiving* information through a traditional one-way information pathway of formal lecture style teaching (33). As Centennials students are savvy with respect to whether information is relevant and of benefit to them in terms of self-development, and if so, they will be self-motivated and engaged in the learning activity and if not the opposite is true (33). As such it is important that educators adapt their approaches to teaching and assessment in line with the needs of the current and evolving generation of students. Giray (32) provides a valuable insight into the characterisation of generations and highlights the valuable advice that “*teachers should teach the students, not the subject.*” It is important that educators understand the current students in terms of learning preferences, styles and digital capabilities to fully adapt and develop pedagogical approaches to teaching and assessment which ensure inclusivity of all learners. As such, continual involvement with the students in a co-productive role when developing the curriculum is a symbiotic relationship to promote successful learning outcomes.

### Student Engagement

Although access to the toolkit was assessed, it must be realised that such access does not necessarily translate to level of engagement but solely relates to participation in a most basic form of access to information and does not address individual understanding (34). As engagement has several meanings, for the purposes of this project, a perspective of student engagement was considered by the access and extent of utilisation of the toolkit, students’ perceptions of the resources, reflective feedback and the successful completion of assessment activities. Access data (Supplementary Figure S2) highlights the importance of introducing new learning resources earlier within the module and providing opportunities such as workshops and tutorials to encourage earlier active engagement rather than students only consulting the resource during the final stages of the submission of their assessments.

### Lay Writing

Quantitative evaluation of scientific abstracts and lay summaries in terms of readability metrics indicated that students were successful at moderating their style of writing for the lay audience as the FRE (ease of readability) for the lay summary was higher and the FKGL (target educational grade) was a lower than their scientific abstract (Table 3). This was comparable to readability metrics from authors of scientific journal articles, highlighting the professionalism with which students prepared these lay writing outputs. It should be noted, however that these parameters do not assess scientific accuracy but writing style and readability.

### The Importance of Reflection

When conducting an action research project which undertakes the introduction of a novel learning activity into a well-established module, involving a large student cohort from several courses and forty academic staff members, it is essential to reflect together to ensure the validity of the innovation. Collective reflections permit the researcher to reflect from all stakeholder perspectives and allow an in-depth critical evaluation to engender and further develop innovative teaching practice at both an individual and institutional level. Such transformative reflection will result in tangible changes rather than just a deeper understanding of current practice (35). This project instilled the importance of the inclusion of the student voice to understand what motivates students to learn, how they learn and the best approach to ensure opportunities are provided to develop skills to learn coupled with skills to successfully gain employment and ultimately provide a valuable civic contribution (36, 37).

**FIGURE 7:**
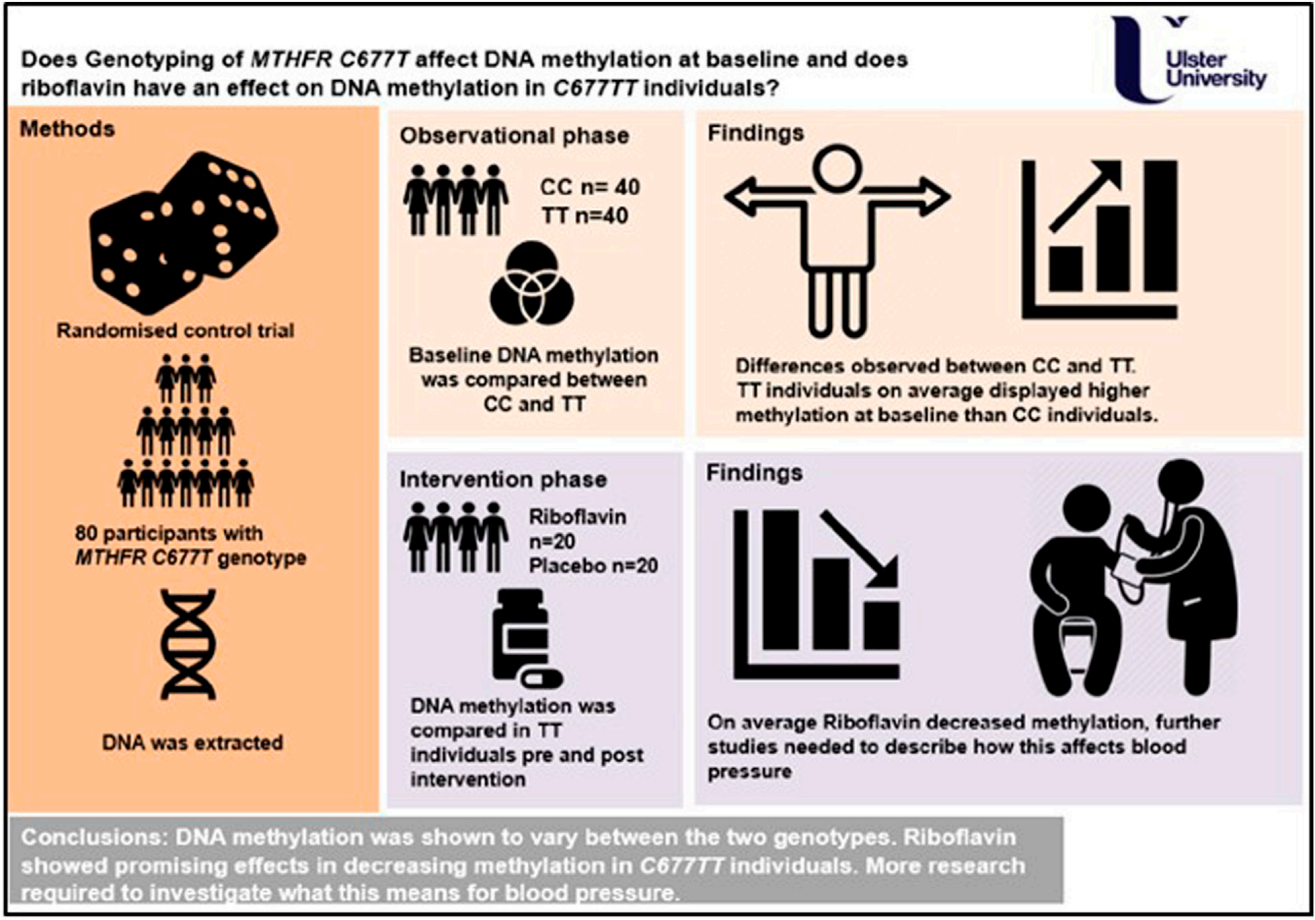
Example of a visual abstract prepared by an undergraduate student studying Biomedical Science.

Personal communications and informal feedback from staff indicated that staff, valued the quality of educational resources provided and classed these activities as valuable additions to the Level 6 curriculum with many hoping they would be a permanent feature. Staff acknowledged the further potential of this innovative initiative, particularly through embedding small aspects of these learning activities in first/second year curricula and expansion to Masters and PhD levels. Staff believed that lay writing was an important addition to the module to develop communication skills with key stakeholders which would be of value when seeking employment as graduates in science, healthcare and non-science careers. It was acknowledged for graduates entering careers as biomedical scientists, the ability to communicate with varied audiences was an important regulatory standard of proficiency. One member of staff, however, felt these activities were beyond the capabilities of undergraduate students, however the standard of work produced by the students as indicated by the readability statistics in relation to the lay summary (Table 3) and the professional creation of visual abstracts demonstrated in Figure 7 evidenced that this was not the case. One member of staff believed that these activities were only of value to students pursuing research careers, however the embedding of lay science communication skills have been successfully introduced into undergraduate degree programmes in other universities (38).

### Reflecting on Creativity and the Development of Skills

Students reflected that they enjoyed the opportunity to be creative when preparing the visual abstracts, with 90.3% of students reporting in the questionnaire that they had the opportunity to be both creative and demonstrate complex problem solving. Creativity is skill valued by employers in all sectors however, it has been reported that while creativity is the pinnacle of Bloom’s cognitive taxonomy, not all Biomedical Science undergraduate degree programmes may necessarily refer to creativity in their curricula nor may students recognise creativity opportunities within the curricula (38). Both staff and students may be unclear as to what is meant by creativity within scientific disciplines and as such do not feel that there are opportunities within scientific degree programmes to be creative nor for students’ creative skills to be acknowledged (39). There are many opportunities in Biomedical Science research activities to be creative and indeed when programmes are analysed it becomes apparent that there are many opportunities within the curricula to encourage creativity which can take many formats and output, e.g., the preparation of review and original scientific papers (39). In this novel visual abstract student assessment activity, students could clearly see the creative opportunity and the opportunity to develop higher order critical evaluation of their investigative projects while utilising and developing their creative and digital skills. One student stated they would use these skills to create learning materials for secondary students when they commenced science teaching training in the next academic year, highlighting the transferability of these skills in different employment pathways of successful biomedical science graduates, both research and non-research alike.

There was a difference between student and staff perspectives in terms of ranking skills development. Skills developed within the undergraduate investigative project, which were viewed highly important by staff but a lower ranking by students were problem solving (staff 93%; students 59%), analytical skills (staff 93%; students 55%), communication (staff 93%; students 69%), independent working (staff 93%; students 79%) and scientific communication, written (staff 93%; students 69%) and oral (staff 93%; students 55%).

Students indicated that time management ranked highly important (83%) in comparison with staff (67%). Staff ranked skills associated with poster preparation very/highly important 93%, however students only 70%. A higher proportion of students than staff ranked very/highly important; digital skills (staff 73%; students 82%) and communicating with audiences with varied levels of understanding (staff 80%; students 86%). It is interesting that students indicated a higher very/highly importance regarding the opportunity to develop innovation skills (staff 60%; students 86%), reflective writing (staff 53%; students 76%), creativity (staff 57%; students 63%) and visual abstracts (staff 67%; students 72%). These findings indicate that although the core assessment outputs of this investigative project module focus on traditional laboratory research skills and scientific communication, students value the opportunity to develop other 21st Century skills and higher order cognitive skills such as creativity.

In any module which is delivered by multiple staff members, it is important to consider all individual staff perspectives, prior to further embedding scientific communication skills and reflective practice within undergraduate degree programmes. Furthermore it is important to share the background and rationale prior to the introduction of such scientific communication initiatives with course teams to highlight i) the importance of lay and visual communication approaches; ii) where such approaches are used in careers within and outside academia and iii) how they differ from the current conventional assessment approaches, e.g., poster presentation, as dismissal of the introduction of novel assessment may result from a lack of understanding of these concepts.

### Embedding Reflective Writing in the Biomedical Science Curriculum

Although students in previous years were encouraged to reflect on their experiences within this final year Investigative Research Project module, many chose not to do so or provided limited reflective reports. It is unknown as to the reason why many students chose not to do so, however two possible reasons include uncertainty regarding how to prepare such a written refection and the fact that the reflective writing was not assessed. Hence, during the current study, resources in the toolkit were included to provide an in-depth guidance on how to optimally reflect for employability in terms of transferable skills developed including digital competencies, and subsequently in this cohort, 93% of students chose to participate in the unassessed reflective writing activity.

Only half of staff ranked reflective writing as an important/very important skill to develop at undergraduate level, even though this is an essential standard of proficiency required of all biomedical scientists and common practice within the varied biomedical science graduate employment sector. It is therefore, essential to seek opportunities to inform and work together with staff on the importance of reflective activities in relation to critical evaluation of personal and others’ capabilities throughout the education experience, as outlined in the SEEC Credit Level Descriptors for Higher Education (40). Further encouragement to embed reflection within the curricula earlier will help students develop how, where and why they learn, which in turn will motivate students providing opportunities to develop competencies related to learning, as well as skills for future use, whether in education or employment (36). The inclusion of written reflection as a form of assessment has been debated; however, it is important that students undertake such activities to develop their personal learning approaches and transferable skills required for future employment (41). Staff may be reluctant to engage students in such reflective activities either assessment or personal reflective logs/diaries, primarily due to lack of knowledge regarding reflection and as such a workshop/shared practice event could be held to highlight the importance of written reflection within the curriculum, styles of reflection and how to successfully reflect (42). Reflection is a key component of the CPD of healthcare professionals and this is embedded within the Standards of Proficiency for Biomedical Scientists, to ensure the continued quality of practice (1).

### Going Forward

Some interesting approaches to further develop and embed these communication and digital skills in the undergraduate programmes were suggested by students and staff in free text responses to the questionnaires and during the reflective focus sessions. Reflections from students have caused the evaluation of the teaching staff’s educational practice which require development, related to Bandura’s social cognitive theory and in particular student self-efficacy, confidence and engagement by including appropriate activities to ensure students reach their personal goals (43). During this study, imposed “*emergency*” distance learning and social implications resultant from the current pandemic impacted on human relationships and interactions which are important in teaching and learning (23).

A return to conventional face-to-face approaches to delivering teaching and the introduction of group-activities will provide opportunities to advance the delivery of teaching through a blended-learning approach. Students indicated that these novel scientific communication skills should be introduced earlier in the degree programmes to enable the continual reinforcement of information and skills development prior to application of these in the final stages of the undergraduate investigative project aligning with Brunner’s spiral curriculum (44). It was also suggested that in future years examples of visual abstracts and lay summaries prepared by students could be provided and students advocated the inclusion of a workshop where students could actively learn and co-prepare these communication outputs, enhancing the learning experience through discussion and collaboration (14). Fifty-three students have subsequently consented to share their outputs, indicating the level of engagement students possessed with these activities and willingness to further support and develop educational experiences for future students. Staff indicated role play could practically develop scientific communication skills to a varied audience. This active method of learning has been used in higher education to foster self-efficacy and confidence in relation to scientific communication (45) driving motivation for learning and ultimately academic attainment (46) and warrants further consideration.

This final year module introduced the concept of reflecting for employability and students’ written reflections highlighted that they appreciated the opportunities to develop both discipline-specific skills, albeit that the technical skills were greatly impacted due to the COVID-19 pandemic, and transferable skills for their future careers. This concurs with Demaria et al. (47) that capstone modules in such biomedical science degrees should embed a focus on such transferable skills. The opportunity to encourage students to undertake written reflection in such modules, however should not be limited to the final year curriculum, rather embedded throughout all levels of the curriculum further enabling students to be aware of employability skills and develop their capabilities and confidence as they progress through such taught programmes and continue within a varied workplace. Such an approach has been incorporated within our institution.

**FIGURE 8:**
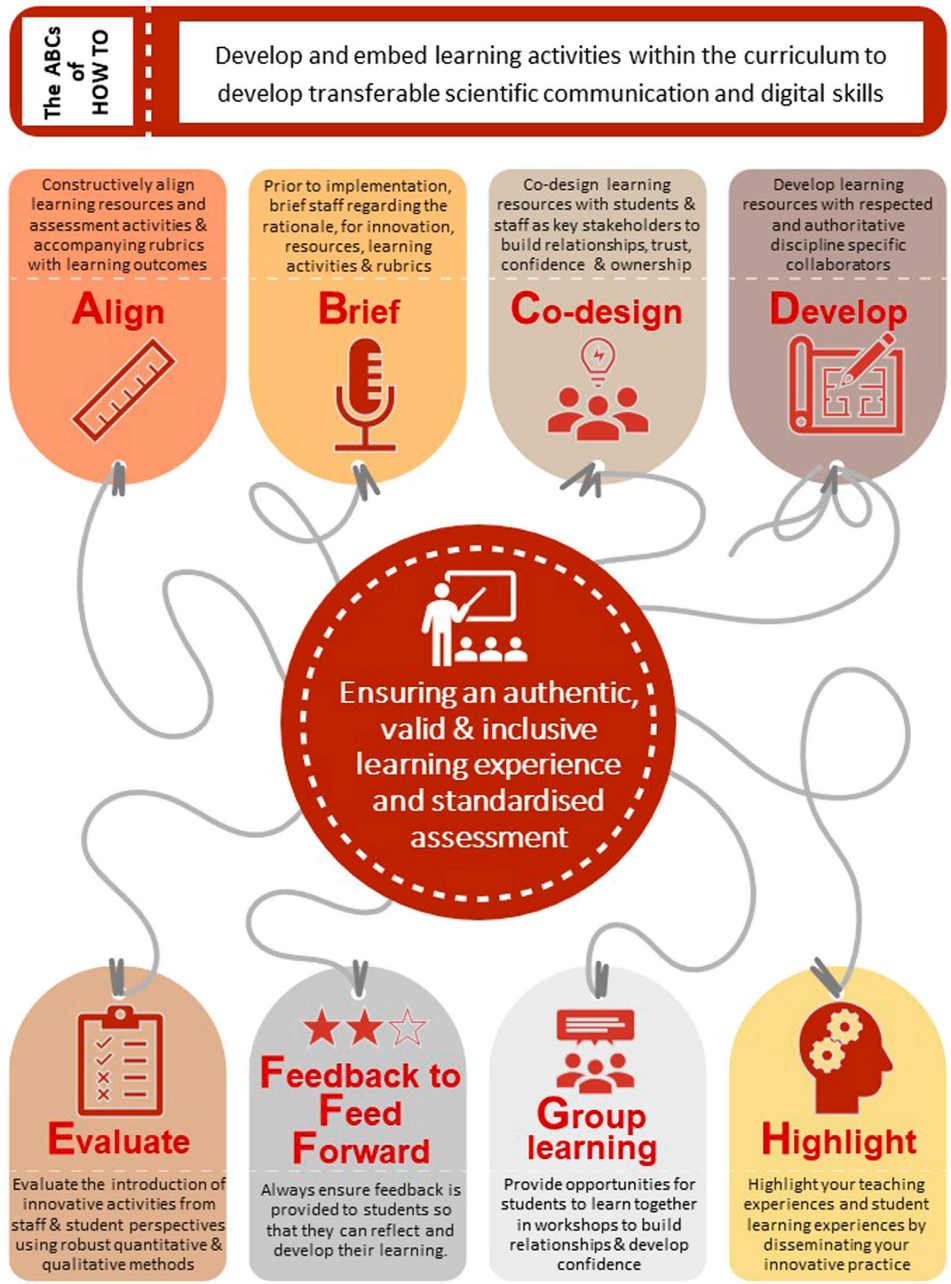
Reflective recommendations on how to embed novel learning activities into the biomedical science curriculum.

Figure 8 offers some further reflective recommendations to others who wish to develop and embed novel learning activities into the biomedical science curriculum.

### Study Limitations

Small focus groups, by design, were used in this project, however, whole-class co-design/co-creation, although a challenge, due to class sizes and multiple supervisors would have enabled even stronger partnerships to be built, ensured inclusiveness and permitted a more democratic contribution to curriculum development (22, 48). The opportunity of students to reflect and complete respective surveys, however, enabled a holistic contribution to further development of teaching and assessment. Whilst an anonymous online survey approach allowed both students and staff the opportunity to be honest in expressing their feelings and assessing the questions in a non-time dependent manner, it has been reported that such online surveys generally only report a 30%–40% uptake rate (49), which was lower in this study, which may potentially result in a non-response related bias. Furthermore, a reason for such an uptake rate could reflect that as this module was the final module of the students’ undergraduate degree programme and invitations to complete the online questionnaire were sent after completion of final modular examinations due to ethical implications to ensure students were not pressurised to complete such questionaries at a time when they were focused on other important study and assessment deadlines. All students, however, were under similar pressures and all students had the same opportunity to complete the questionnaires. Completed questionnaires were received from students enrolled in all degree programmes and as such reflected the views of all student cohorts. Similarly, staff were under pressure with work commitments associated with examination boards. As such it must be considered that the survey data is more of a snapshot of the two populations rather than a total population.

## Conclusion

In conclusion, the co-designed and co-created toolkit resulted in an informative and valued resource by both staff and students. Successful engagement by students, with the resource, particularly the sections relating to practical guidance, resulted in scientific communication outputs which were of comparable standards to professional scientific authors, as evidenced by the readability analysis of students’ work. Students reported, during the reflective focus groups and in the free text responses in the survey, that the activities were enjoyable and as such empowered them to prepare creative outputs which also enabled a large proportion of staff to assess the skills which students had developed, as well as an increased understanding of the significance of the research conducted. Students’ written and focus group reflections and questionnaire responses highlighted the capabilities which they developed and used with confidence to prepare outputs which they felt were accomplished and proud of and students acknowledged the value of developing such transferable communication and digital skills for future use in various employment sectors.

## Summary Table

### What is Known About the Subject?

• The HCPC revised standards of proficiency (SoP) for biomedical scientist registrants, are effective from 1 September 2023
• SoPs place greater emphasis on registrants using information, communication and digital technologies to communicate effectively
• QAA (2023) Biomedical Science Benchmark statement promotes authentic assessment of communication to scientific and lay audiences

### What This Paper Adds

• Co-creation of a scientific communication toolkit enhanced student engagement and support of authentic assessments
• Readability metrics demonstrated an ability to moderate writing for the lay community
• The preparation of visual abstracts encouraged creativity, critical appraisal and development of digital communication skills

## Summary Sentence

This work represents an advance in biomedical science because authentic scientific communication assessment promoted the development of key transferrable digital and communication skills for future employment.

## Data Availability

The datasets presented in this article are not readily available per ethics approval. Further enquiries should be directed to bcmillar@niphl.dnet.co.uk.
