# Supplementary material for: Embedding Scientific Communication and Digital Capabilities in the Undergraduate Biomedical Science Curriculum
Source: Br J Biomed Sci. 2023 Apr 19;80:11284. doi: 10.3389/bjbs.2023.11284 (PMC10154515; doi:10.3389/bjbs.2023.11284)
Supplement: Supplementary file 1 [file DataSheet6.PDF]

Supplementary 6

Statistical analysis of lay writing versus courses

Statistical analysis using a one way ANOVA to examine whether there are differences in (i) Flesch Reading Ease Score and (ii) Flesch Kincaid Grade Level (for the Lay and the Scientific writing) between students enrolled in different courses within the school i.e. Biology (n=24), BMS 3y (n=79), BMS DPP Path (20), BMS DPP (n=23) i.e. 146 students' data in total.

No significant differences, per 'Sig.' column i.e.. no significant difference between courses

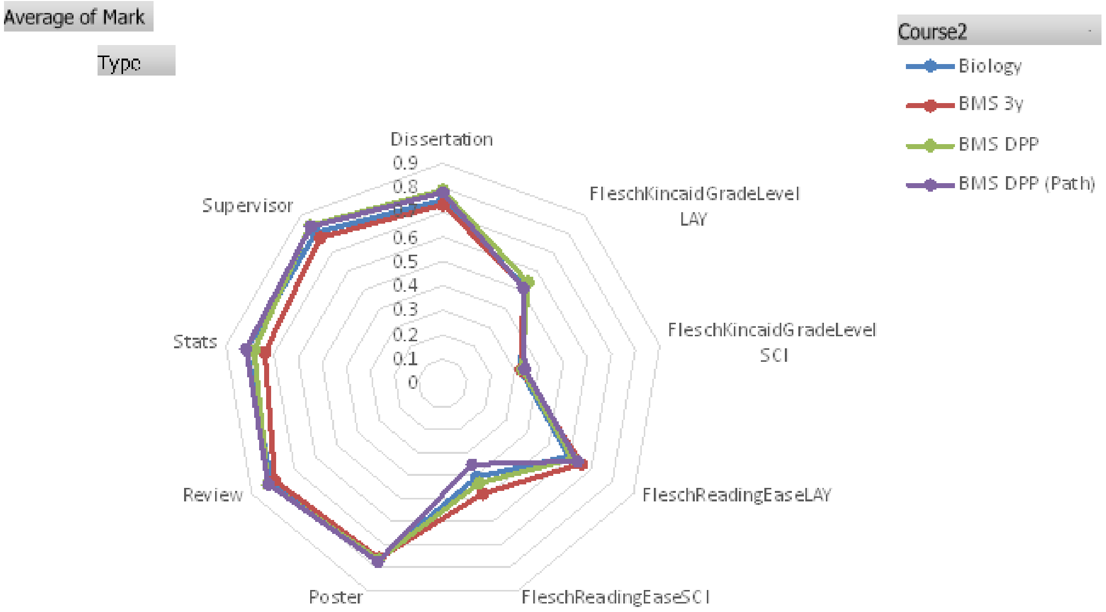

ANOVA						
		Sum of Squares	df	Mean Square	F	Sig.
Flesch Reading Ease (SCI)	Between Groups	644.271	3	214.757	1.127	0.340
	Within Groups	27052.032	142	190.507		
	Total	27696.303	145			
Flesch-Kincaid Grade Level (SCI)	Between Groups	10.713	3	3.571	0.258	0.856
	Within Groups	1968.774	142	13.865		
	Total	1979.487	145			
Flesch Reading Ease (LAY)	Between Groups	454.287	3	151.429	0.948	0.419
	Within Groups	22671.831	142	159.661		
	Total	23126.118	145			
Flesch-Kincaid Grade Level (LAY)	Between Groups	5.589	3	1.863	0.369	0.775
	Within Groups	716.551	142	5.046		
	Total	722.140	145			
