# Supplementary material for: Embedding Scientific Communication and Digital Capabilities in the Undergraduate Biomedical Science Curriculum
Source: Br J Biomed Sci. 2023 Apr 19;80:11284. doi: 10.3389/bjbs.2023.11284 (PMC10154515; doi:10.3389/bjbs.2023.11284)

Supplementary Figure 5

Students (n=31) preferences on how information and guidance should be delivered.

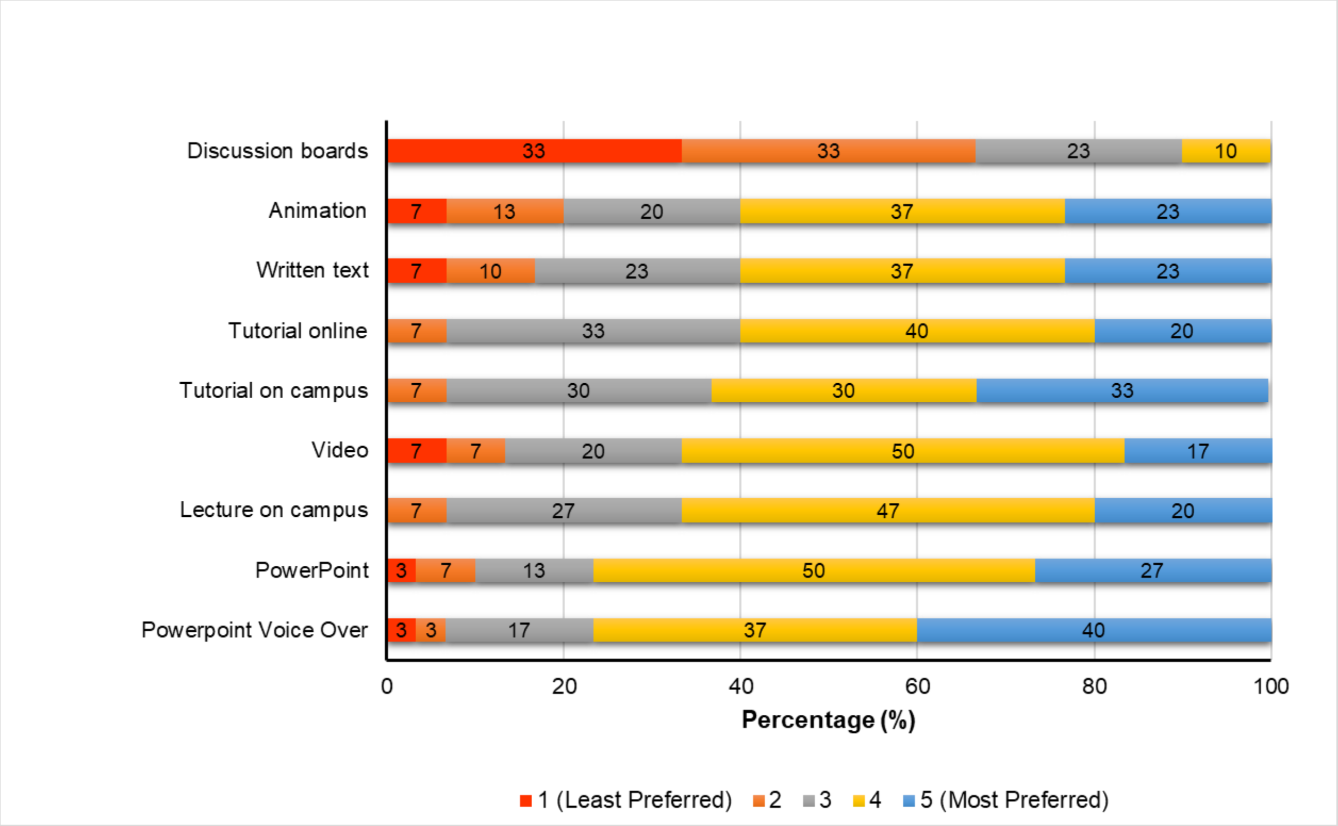

Supplement: Supplementary file 2 [file Image5.pdf]
