# Supplementary material for: Embedding Scientific Communication and Digital Capabilities in the Undergraduate Biomedical Science Curriculum
Source: Br J Biomed Sci. 2023 Apr 19;80:11284. doi: 10.3389/bjbs.2023.11284 (PMC10154515; doi:10.3389/bjbs.2023.11284)
Supplement: Supplementary file 3 [file Table1.pdf]

Supplementary Table 1: Student Questionnaire

BMS505 Scientific Communication & Digital Literacy

This survey is part of an Action Research Project promoting "Scientific Communication" and "Digital Literacy" within the final year BMS505 Investigative Project. Ulster University. The purpose of the survey is to explore student awareness, behaviours and perceptions of (i) the support provided to help students complete their assignments during the module, (ii) the skills acquired and (iii) the transferable employability skills developed.

This survey is being conducted by Professor Cherie Millar and Dr. Nigel Ternan (School of Biomedical Sciences) as part of an Action Research Project as part of an MEd programme of study.

Your participation in this survey is completely voluntary. You may choose not to participate. If you decide to participate in the survey, you may withdraw at any time. Participation or non-participation in this survey will not be linked in anyway to your awarded mark for the module.

This online survey will take approximately 15-20 minutes to complete, and before you start the survey you will be asked to if you give consent to participate in this study and for Ulster University to use your non-identifiable data.

To help protect your confidentiality, the surveys will not contain information that will personally identify you and as such will be completely anonymous. All anonymised data will be in stored accordance with Ulster University's General Data Protection Policy (GDPR). Information on the Ulster University's GDPR complaints procedure can be obtained at https://www.ulster.ac.uk/about/governance/compliance/gdpr

This study has been approved by the Centre for Higher Education and Research Practice (CHERP), Ethics Filter Committee. This research has been reviewed according to Ulster University procedures for research involving human subjects.

If you have any questions about the survey, please contact Professor Cherie Millar (c.millar@ulster.ac.uk).

1. ELECTRONIC CONSENT: Please select your choice below.

Clicking on the "agree" button below indicates that:

- you have read the above information
- you voluntarily agree to participate

If you do not wish to participate in this research study, please decline participation by clicking on the "disagree" button.

○ Agree

○ Disagree

2. Gender

○ Female

○ Male

○ Other

○ Prefer not to say

3. Which UG degree programme are you undertaking?

○ BSc Hons Biomedical Science

○ BSc Hons Biomedical Science DPP

○ BSc Hons Biomedical Science DPP(Path)

○ BSc Hons Biology

Section 1: Capabilities

Please rank your capabilites at various stages within BMS505- Investigative project

Where 1=strongly disagree; 2= disagree; 3= neutral; 4=agree; 5=strongly agree

4. I have demonstrated complex problem solving during BMS505

1 2 3 4 5

5. I have developed creativity during BMS505

1 2 3 4 5

Section 2: Searching and Analysing the Sceintific Literature

Please rank your capabilities where 1=poor; 2=limited; 3=adequate; 4=good; 5=excellent

6. SEARCHING THE SCIENTIFIC LITERATURE at the START of the module.

1 2 3 4 5

7. SEARCHING THE SCIENTIFIC LITERATURE at the END of the module.

1 2 3 4 5

8. READING & ANALYSING THE SCIENTIFIC LITERATURE at the START of the module.

1 2 3 4 5

9. READING & ANALYSING THE SCIENTIFIC LITERATURE at the END of the module.

1 2 3 4 5

Section 3: Preparation of Abstracts

Please rank your capabilities where 1=poor; 2=limited; 3=adequate; 4=good; 5=excellent

10. WRITING SCIENTIFIC FINDINGS IN A LAYMAN SUMMARY/ABSTRACT at the START of the module.

1 2 3 4 5

11. WRITING SCIENTIFIC FINDINGS IN A LAYMAN SUMMARY/ABSTRACT at the END of the module.

1 2 3 4 5

12. PREPARING A VISUAL ABSTRACT at the START of the module.

1 2 3 4 5

13. PREPARING A VISUAL ABSTRACT at the END of the module.

1 2 3 4 5

14. PREPARING A SCIENTIFIC ABSTRACT at the START of the module.

1 2 3 4 5

15. PREPARING A SCIENTIFIC ABSTRACT at the END of the module.

1 2 3 4 5

Section 3: Preparing a witten dissertation and poster

Please rank your capabilities where 1=poor; 2=limited; 3=adequate; 4=good; 5=excellent

16. PREPARING WRITTEN DISSERTATION IN THE STYLE OF A SCIENTIFIC PAPER at the START of the module

1 2 3 4 5

17. PREPARING WRITTEN DISSERTATION IN THE STYLE OF A SCIENTIFIC PAPER at the END of the module

1 2 3 4 5

18. PREPARING A SCIENTIFIC POSTER at the START of the module

1 2 3 4 5

19. PREPARING A SCIENTIFIC POSTER at the END of the module

1 2 3 4 5

Section 4: Support

Please rank how the resources provided supported you to complete the various scientific communication tasks throughout the module
1=poor; 2=limited; 3=adequate; 4=good; 5=excellent

20. Please rank how the resources provided in BMS505 supported you to complete the various scientific communication tasks throughout the module
1=poor; 2=limited; 3=adequate; 4=good; 5=excellent

	1	2	3	4	5
Searching the literature					
Read & analyse the literature					
Prepare a scientific abstract					
Prepare a visual abstract					
Prepare a layman summary/abstract					
Prepare a written dissertation in the style of a scientific paper					
Prepare a poster					

21. Do you feel the Elsevier Support enhanced your learning experience?

1=not at all; 2=limited; 3=not sure; 4=good support; 5=excellent support

1 2 3 4 5

22. Please rank how useful you found the resources within the Elsevier Research Academy.

1=poor; 2=limited; 3=adequate; 4=good; 5=excellent

1 2 3 4 5

23. Which of the Elsevier Research Academy modules did you find useful?

Enter your answer

24. Did you complete and obtain a certificate for participation in any of the Elsevier Research Academy modules? If yes please state which ones.

Enter your answer

25. Please list any further resources/support topics which could have been provided within BMS505 you feel would have helped you to successfully complete your assignments.

Enter your answer

26. What is your preference in relation to how information and guidance is delivered.

Where 1 is least prefered and 5 is most prefered

	1	2	3	4	5
Written text					
Video					
Animation					
Powerpoint slides					
Powerpoint voice over					
Tutorial on campus					
Tutorial on-line					
Discussion Boards					
Lecture on campus					

Section 5: Development of digital skills

During this module you have had the opportunity to develop digital literacy skills as you completed your assignment tasks.

27. Please rank the respective areas in relation to your development of digital literacy during BMS505 where 1= least developed and 5= highest developed

	1	2	4	5
Information retrieval (papers & data)				
Data analysis (software & presentation)				
Communication (scientific)				
Communication (General)				
Collaboration				
Technical Proficiency (MS applications Word,Powerpoint, Excel)				
Technical Proficiency (Statistical tools)				
Creation using digital tools (Visual abstract)				
Creation using digital tools (Poster)				
Expanding knowledge (e.g. Elsevier Research Academy, on-line resources)				

28. What are your career plans after completing your undergraduate degree?

Enter your answer

29. Please list any skills which you have developed throughout this module which you believe are transferable within your future workplace environment

Enter your answer

30. Please give examples of how you will use these skills in your future post-graduate studies or employment.

Enter your answer

31. In your opinion, how important is it that students studying Biomedical Science at undergraduate level have the oportunity to develop the following skills:-
Where 1=not important and 5 is highly important

	1	2	3	4	5
Communication	○	○	○	○	○
Collaboration	○	○	○	○	○
Scientific Communication (written)	○	○	○	○	○
Scientific Communication (oral)	○	○	○	○	○
Scientific Communication to individuals of different levels of understanding	○	○	○	○	○
Scientific communication visual abstract	○	○	○	○	○
Creative	○	○	○	○	○
Reflective Writing	○	○	○	○	○
Analytical	○	○	○	○	○
Problem Solving	○	○	○	○	○
Digital	○	○	○	○	○
Time Management	○	○	○	○	○
Independent working	○	○	○	○	○
Innovation	○	○	○	○	○
Scientific communication poster	○	○	○	○	○

32. Please clasify how confident you would be in applying the skills which you have developed during this module in your future studies and career
Where 1=not confident and 5 is highly confident

	1	2	3	4	5
Communication	○	○	○	○	○
Collaboration	○	○	○	○	○
Scientific Communication (written)	○	○	○	○	○
Scientific Communication (oral)	○	○	○	○	○
Scientific Communication to individuals of different levels of understanding	○	○	○	○	○
Scientific communication (visual abstract)	○	○	○	○	○
Creative	○	○	○	○	○
Reflective Writing	○	○	○	○	○
Analytical	○	○	○	○	○
Problem Solving	○	○	○	○	○
Digital	○	○	○	○	○
Time Management	○	○	○	○	○
Independent working	○	○	○	○	○
Innovation	○	○	○	○	○
Scientific Communication (poster)	○	○	○	○	○

33. Please use this opportunity to express any other comments you may have in relation to scientific communication and digital skills

Enter your answer
