# Supplementary material for: Embedding Scientific Communication and Digital Capabilities in the Undergraduate Biomedical Science Curriculum
Source: Br J Biomed Sci. 2023 Apr 19;80:11284. doi: 10.3389/bjbs.2023.11284 (PMC10154515; doi:10.3389/bjbs.2023.11284)
Supplement: Supplementary file 4 [file Table2.pdf]

Supplementary Table 2: Staff questionnaire

Staff Evaluation Questionnaire relating to Scientific Communication & Digital Literacy

This survey is part of an Action Research Project promoting "Scientific Communication" and "Digital Literacy" within the final year BMS505 Investigative Project. Ulster University. The purpose of the survey is to explore student awareness, behaviours and perceptions of (i) the support provided to help students complete their assignments during the module, (ii) the skills acquired and (iii) the transferable employability skills developed.

This survey is being conducted by Professor Cherie Millar and Dr. Nigel Ternan (School of Biomedical Sciences) as part of an Action Research Project as part of an MEd programme of study.

You are invited to participate in this action research project because you are currently a member of staff supervising students enrolled in BMS505 Investigative Project Module (2020-21). This means you are a key stakeholder in the development of resources to support students and you will have experience of how such activities and resources have helped students to develop their transferrable scientific communication and digital skills for use in higher education and employment.

Your participation in this survey is completely voluntary. You may choose not to participate. If you decide to participate in the survey, you may withdraw at any time.

This online survey will take approximately 15 minutes to complete, and before you start the survey you will be asked to if you give consent to participate in this study and for Ulster University to use your non-identifiable data.

To help protect your confidentiality, the surveys will not contain information that will personally identify you and as such will be completely anonymous. All anonymised data will be in stored accordance with Ulster University's General Data Protection Policy (GDPR). Information on the Ulster University's GDPR complaints procedure can be obtained at https://www.ulster.ac.uk/about/governance/compliance/gdpr

This study has been approved by the Centre for Higher Education and Research Practice (CHERP), Ethics Filter Committee. This research has been reviewed according to Ulster University procedures for research involving human subjects.

If you have any questions about the survey, please contact Professor Cherie Millar (c.millar@ulster.ac.uk).

1. ELECTRONIC CONSENT: Please select your choice below.

Clicking on the "agree" button below indicates that:

• you have read the above information
• you voluntarily agree to participate

If you do not wish to participate in this research study, please decline participation by clicking on the "disagree" button.

○ Agree

○ Disagree

2. In your opinion, how important is it that students studying Biomedical Science at undergraduate level have the opportunity to develop the following skills:-
Where 1=not important and 5 is highly important

	1	2	3	4	5
Communication	○	○	○	○	○
Collaboration	○	○	○	○	○
Scientific Communication (written)	○	○	○	○	○
Scientific communication (oral)	○	○	○	○	○
Scientific communication to indiviudals of different levels of understanding	○	○	○	○	○
Scientific communication (visual abstract)	○	○	○	○	○
Creative	○	○	○	○	○
Reflective writing	○	○	○	○	○
Analytical	○	○	○	○	○
Problem solving	○	○	○	○	○
Digital	○	○	○	○	○
Time management	○	○	○	○	○
Independent working	○	○	○	○	○
Innovation	○	○	○	○	○
Scientific communication poster	○	○	○	○	○

3. In BMS505 students were asked to complete a Visual Abstract to summarise their research findings.

How applicable did you feel this task was to careers which students may pursue as graduates?

Where 1= not applicable and 5 is extremely applicable

1 ○ 2 ○ 3 ○ 4 ○ 5 ○

10. Did the Lay Summary submitted by the students which you supervised help in the assessment of the skills you descibed in the previous question?

Where 1= not at all and 5 is extremely helpful

1 ○ 2 ○ 3 ○ 4 ○ 5 ○

11. Did you read the Lay Summary included in the dissertations of students which you did not supervise but marked?

○ Yes

○ No

12. Did the lay summary submitted by the students whose dissertations you assessed help in your overall understanding of their investigative project?

Where 1= not at all and 5 is extremely helpful

1 ○ 2 ○ 3 ○ 4 ○ 5 ○

13. Please use this opportunity to provide feedback on the inclusion of these two new scientific communication tasks ie., a visual abstract and a lay summary, within BMS505?

Enter your answer

4. In your opinion what skills did students develop when preparing the Visual Abstract?

Enter your answer

5. Did the visual abstract submitted by the students who you supervised help in your overall assessment of their skills as detailed in the answer above?

Where 1= not at all and 5 is extremely helpful

1 ○ 2 ○ 3 ○ 4 ○ 5 ○

6. Did you read the Visual Abstracts included in the dissertations of students which you did not supervise but marked?

○ Yes

○ No

7. Did the visual abstract submitted by the students whose dissertations you assessed help in your overall understanding of their investigative project?

Where 1= not at all and 5 is extremely helpful

1 ○ 2 ○ 3 ○ 4 ○ 5 ○

8. In BMS505 students were asked to complete a Lay Summary to summarise their research findings.

How applicable did you feel this task was to careers which students may pursue as graduates?

Where 1= not applicable and 5 is extremely applicable

1 ○ 2 ○ 3 ○ 4 ○ 5 ○

9. In your opinion what skills did students develop when preparing the Lay Summary?

Enter your answer
