# Supplementary material for: Embedding Scientific Communication and Digital Capabilities in the Undergraduate Biomedical Science Curriculum
Source: Br J Biomed Sci. 2023 Apr 19;80:11284. doi: 10.3389/bjbs.2023.11284 (PMC10154515; doi:10.3389/bjbs.2023.11284)

Supplementary Figure 4

Students'(n=31) perception of support provided by resources in relation to the investigative project module

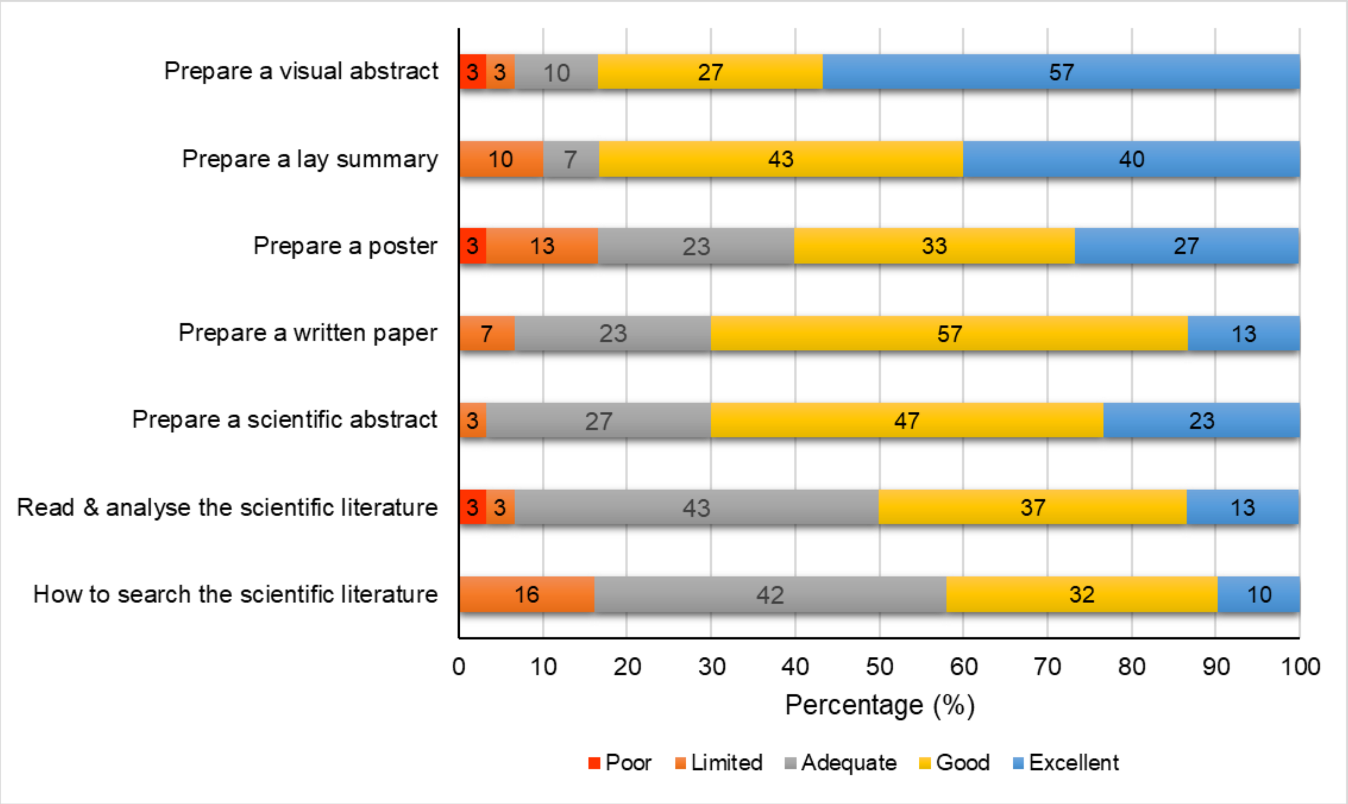

Supplement: Supplementary file 5 [file Image4.pdf]
