# Supplementary figures and images for: Embedding Scientific Communication and Digital Capabilities in the Undergraduate Biomedical Science Curriculum
Source: Br J Biomed Sci. 2023 Apr 19;80:11284. doi: 10.3389/bjbs.2023.11284 (PMC10154515; doi:10.3389/bjbs.2023.11284)

## Supplementary Figure 2

Details when students (n=148) first accessed the toolkit

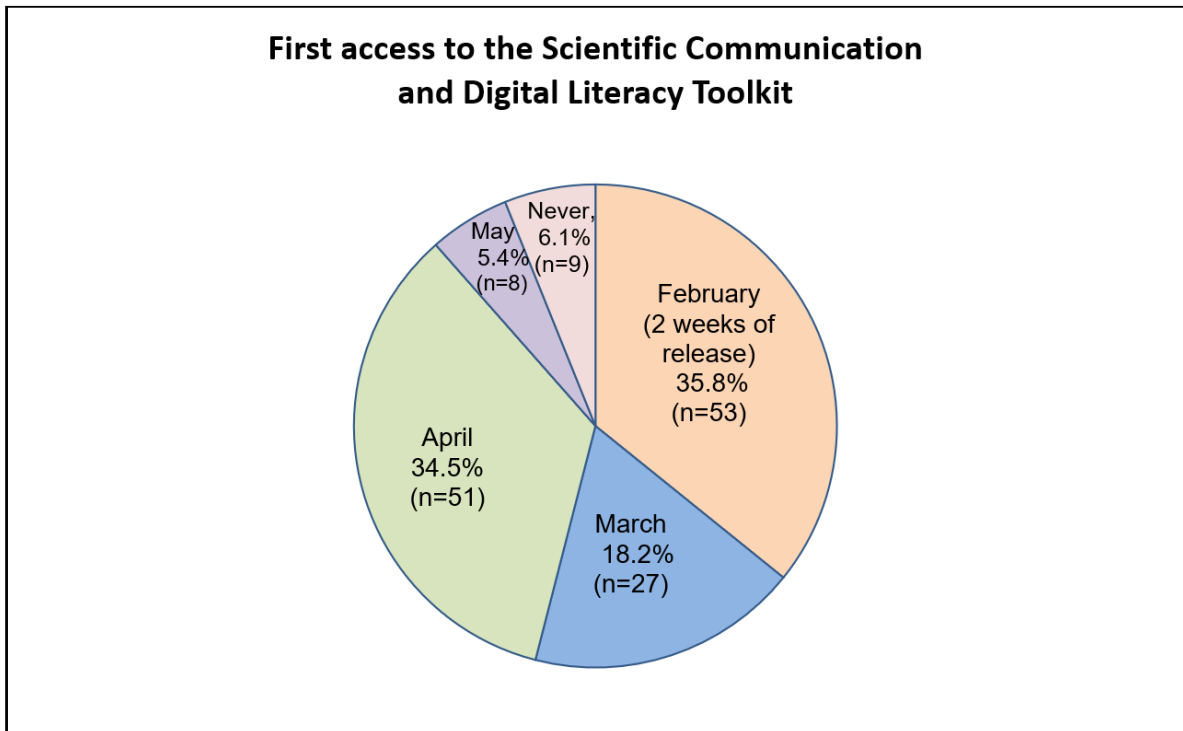

Supplement: Supplementary file 6 [file Image2.pdf]
