# Supplementary material for: Embedding Scientific Communication and Digital Capabilities in the Undergraduate Biomedical Science Curriculum
Source: Br J Biomed Sci. 2023 Apr 19;80:11284. doi: 10.3389/bjbs.2023.11284 (PMC10154515; doi:10.3389/bjbs.2023.11284)
Supplement: Supplementary file 7 [file Image3.pdf]

Supplementary Figure 3

Access to toolkit folders

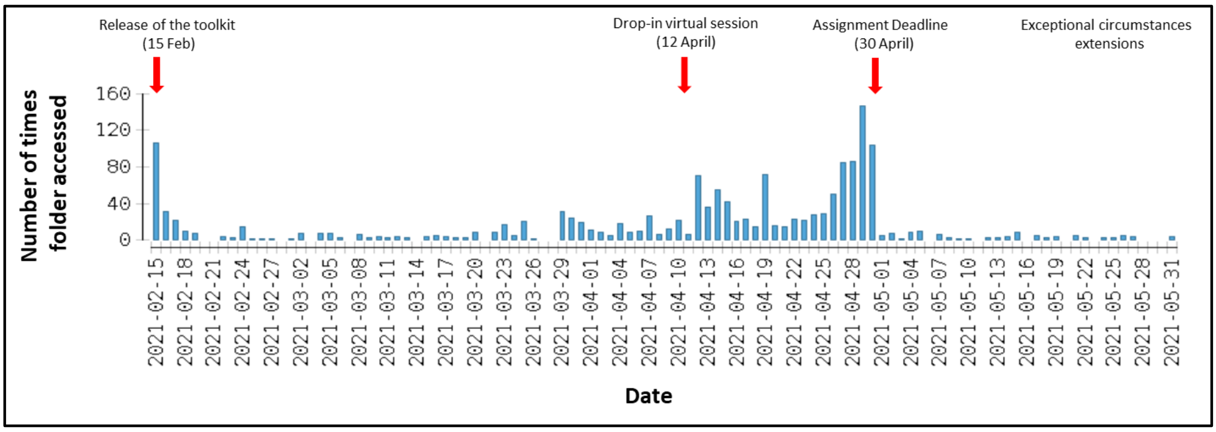

A: Access to lay summary folder

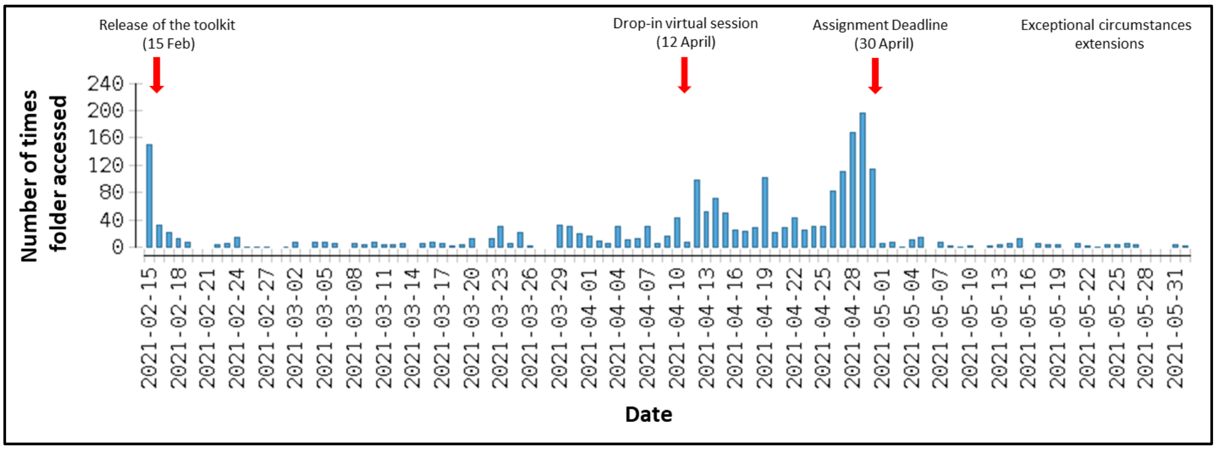

B: Access to visual abstract folder

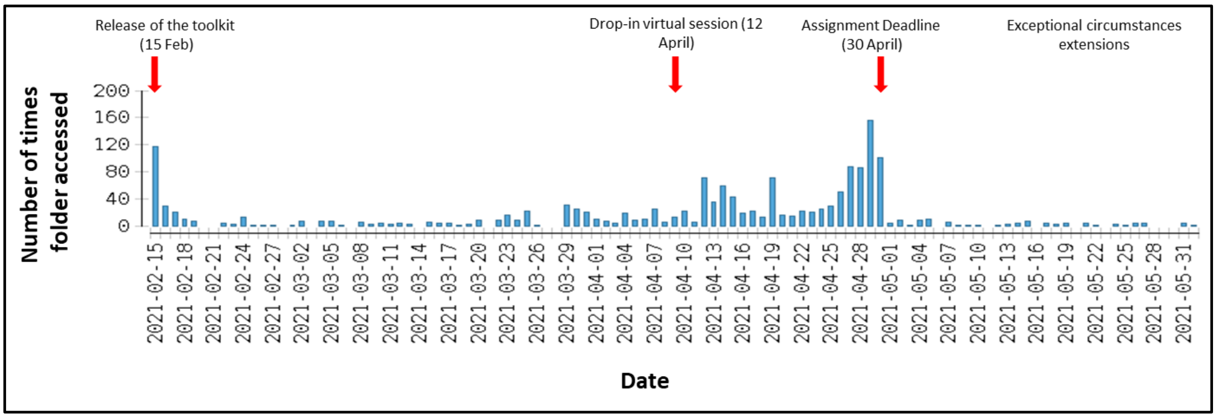

C: Access to transferable skills folder
