# Supplementary material for: Embedding Scientific Communication and Digital Capabilities in the Undergraduate Biomedical Science Curriculum
Source: Br J Biomed Sci. 2023 Apr 19;80:11284. doi: 10.3389/bjbs.2023.11284 (PMC10154515; doi:10.3389/bjbs.2023.11284)
Supplement: Supplementary file 8 [file Table3.pdf]

Supplementary Table 3: Demographics of student respondents to questionnaire

Course	Male (n)	Female (n)	Total
**BSc(Hons) Biology**	1	3	4
**BSc(Hons) Biomedical Science**	6	12	18
**BSc(Hons) Biomedical Science DPP**	2	2	4
**BSc(Hons) Biomedical Science DPP (Path)**	1	4	5
**Total**	**10**	**21**	**31**
