# Supplementary material for: Embedding Scientific Communication and Digital Capabilities in the Undergraduate Biomedical Science Curriculum
Source: Br J Biomed Sci. 2023 Apr 19;80:11284. doi: 10.3389/bjbs.2023.11284 (PMC10154515; doi:10.3389/bjbs.2023.11284)
Supplement: Supplementary file 10 [file Image1.pdf]

Supplementary Figure 1

Key online resources included in the Scientific Communication and Digital Capabilities Toolkit

"The Scientific Communication and Digital Capabilities Toolkit"
Key Online Resources

**Scientific communication**		
Comedy sketch regarding lay communication	YouTube (Alexander Armstrong)	https://youtu.be/3wHKBavY_h8
**Lay writing**		
How to write a lay summary	JISC document (Monica Duke)	https://www.dcc.ac.uk/sites/default/files/documents/publications/HowToLaySummariesDec2012.pdf
Protocol for writing lay summaries	Article	Wada, M. *et al.* A protocol for co-creating research project lay summaries with stakeholders: guideline development for Canada's AGE-WELL network. *Res Involv Engagem (*2020) 6:22. https://doi.org/10.1186/s40900-020-00197-3
Plain Language Summaries (PLS) of Publications Toolkit	Envision Pharma Group	https://www.envisionthepatient.com/plstoolkit/
Plain English Campaign	Guide	http://www.plainenglish.co.uk/free-guides.html
**Visual Abstract**		
How to prepare a visual, by Michelle Lim	Short Course	http://people.duke.edu/~ms203/#/
The Role of Visual Abstracts in the Dissemination of Medical Research	Article	Millar BC, Lim M. The Role of Visual Abstracts in the Dissemination of Medical Research. *Ulster Med J.* (2022) 91(2):67-78. https://www.ums.ac.uk/umj091/091(2)067.pdf
**Transferable Skills**		
Transferable Skills - What Are They and How Can you Develop Them?	YouTube KISSS Career Coaching	https://youtu.be/AOC5kt9mtTg
What are Digital Capabilities?	YouTube University of Derby.	https://youtu.be/ZK9K_a0fq5o
What is Digital capability?	JISC	https://digitalcapability.jisc.ac.uk/what-is-digital-capability/
Hard Skills vs Soft Skills	GCFLearnFree.org	https://youtu.be/0FFLFcB9xfQ
What Are 21st Century Skills?	AES 21st Century Classroom	https://youtu.be/QYfCEnTmr5o
**Reflection**		
Reflection Toolkit	On-line resource University of Edinburgh	https://www.ed.ac.uk/reflection
