# Supplementary material for: Embedding Scientific Communication and Digital Capabilities in the Undergraduate Biomedical Science Curriculum
Source: Br J Biomed Sci. 2023 Apr 19;80:11284. doi: 10.3389/bjbs.2023.11284 (PMC10154515; doi:10.3389/bjbs.2023.11284)
Supplement: Supplementary file 9 [file Image7.pdf]

Supplementary Figure 7

Statistical analysis of writing lay and scientific abstracts and marks awarded

Statistical analysis to examine if there is a correlation between

(i) Flesch Reading Ease Score and (ii) Flesch Kincaid Grade Level with marks (Review, dissertation, supervisor mark, Stats Test, poster) again for the lay and scientific writing.

Summary

- Strong negative correlations between reading ease and respective grade level
- Positive correlations between:
  - scientific and lay scores and reading eases
  - The marks for the components of the project, with review, dissertation, poster correlating strongly
- Small but significant negative correlations between the lay grade level and the marks for the project components (yellow highlight)
- Significant negative correlation between the scientific reading ease and the supervisor mark (orange highlight)

Pearson Correlation									
	Flesch Reading Ease (SCI)	Flesch-Kincaid Grade Level (SCI)	Flesch Reading Ease (LAY)	Flesch-Kincaid Grade Level (LAY)	Review	Dissertation	Supervisor	Stats	Poster
Flesch Reading Ease (SCI)	1.000	-.832**	.335**	-.270**	-0.100	0.019	-.187*	-0.005	-0.013
Flesch-Kincaid Grade Level (SCI)	-.832**	1.000	-.170*	.225**	-0.065	-0.152	0.109	-0.108	-0.097
Flesch Reading Ease (LAY)	.335**	-.170*	1.000	-.881**	-0.005	0.145	0.104	-0.024	0.062
Flesch-Kincaid Grade Level (LAY)	-.270**	.225**	-.881**	1.000	-.172*	-.263**	-.202*	-0.067	-.231**
Review	-0.100	-0.065	-0.005	-.172*	1.000	.663**	.442**	.211*	.540**
Dissertation	0.019	-0.152	0.145	-.263**	.663**	1.000	.407**	.234**	.661**
Supervisor	-.187*	0.109	0.104	-.202*	.442**	.407**	1.000	0.162	.435**
Stats	-0.005	-0.108	-0.024	-0.067	.211*	.234**	0.162	1.000	.247**
Poster	-0.013	-0.097	0.062	-.231**	.540**	.661**	.435**	.247**	1.000
** Correlation is significant at the 0.01 level (2-tailed).									
* Correlation is significant at the 0.05 level (2-tailed).									
